# A novel diagnostic algorithm for chronic and subacute cough

**DOI:** 10.1186/2049-6958-9-33

**Published:** 2014-06-07

**Authors:** Peng Lu, Dan Zhou, Chengzhen Jin

**Affiliations:** 1 Respiratory Department, Second Affiliated Hospital of Harbin Medical University, Harbin 150086, China; 2 Respiratory Department, Fourth Affiliated Hospital of Harbin Medical University, No. 37 Yiyuan Street Nangang District, Harbin 150086, China

**Keywords:** Asthma, Cough, Diagnosis, Gastroesophageal reflux disease, Postnasal drip syndrome

## Abstract

**Background:**

Cough remains the most common reason for patients to seek medical attention. We practised a novel diagnostic algorithm for chronic and subacute cough.

**Methods:**

Chronic and subacute cough patients with normal chest X-ray results and without respiratory tract infections in the preceding eight weeks were recruited. The patients were divided into two groups: Group A, patients with typical symptoms and signs of postnasal drip syndrome (PNDS), asthma syndromes (AS) and gastroesophageal reflux disease (GERD); Group B, patients without the typical symptoms and signs. The two groups received targeted or sequential empirical trials of therapy according to the algorithm.

**Results:**

Among the 524 patients available for analysis in Groups A and B, 436 (83.6%) were diagnosed to have PNDS (34.2%), AS (44.5%) and/or GERD (10.1%), among which 26 had two causes (6.0%) and 6 had three causes (1.4%). After empirical trials of therapy, 81.5% of the patients were diagnosed. The mean time for diagnosis was considerably shorter in Group A (13.1 ± 5.6 d) than in Group B (23.4 ± 7.2 d) (p < 0.01). The diagnosis rate of the first trial in Group A (54.1%) was significantly higher than that in Group B (28.6%, p < 0.01).

**Conclusions:**

The proposed algorithm is a promising and practical approach to diagnose chronic and subacute cough.

## Background

Cough remains the most common reason for patients to seek medical attention [1,2]. Chronic cough is a common and frequently debilitating symptom that is often viewed as an intractable problem [3]. Previous studies have provided many recommendations on how to manage chronic cough. They paid more attention to the objective tests [4,5]. However, the problem is the low availability, poor efficacy, and high cost of some major diagnostic tests [6-12] in developing countries, such as in China. In addition, in case of patients for whom a specific etiology of chronic cough is not apparent, ACCP guideline said empiric therapy for UACS in the form of a first-generation A/D preparation should be prescribed before beginning an extensive diagnostic workup [13]. Many studies have suggested that gastroesophageal reflux disease (GERD) be first treated empirically in patients with chronic cough according to their typical symptoms and signs [14] or after ruling out postnasal drip syndrome (PNDS), cough- variant asthma (CVA), and eosinophilic bronchitis (EB) [3,11,15,16]. Therefore, current investigations must be further refined, and more emphasis should be placed on the diagnostic function of empirical trials of therapy [9,10,16]. In this study, we set up a novel and practical diagnostic algorithm and prospectively evaluated its feasibility.

## Methods

### Patient population

We consecutively studied immunocompetent patients (age: 18 to 72 years) who were referred to our university outpatient clinic between January 2005 and October 2011 for the evaluation of chronic cough (more than 8 weeks), and subacute cough (≥ 3 weeks but ≤ 8 weeks). This study included patients with normal chest X-ray results and without a previous history of heart diseases and respiratory tract infections in the preceding eight weeks, bloody sputum, obvious long term or relapsing purulent sputum and nasal and postnasal purulent discharge. This study was conducted in accordance with the declaration of Helsinki and with approval of the Ethics Committee of Harbin Medical University. Written informed consent was obtained from all participants. The registration number of the prospective clinical trial is ChiCTR-ONRC-10001130.

### Diagnostic algorithm

The history of the patients was carefully reviewed, and physical examination was performed. Patients with post-infectious cough and heart diseases, as well as patients with the highest cough score (frequency plus severity) [11] of less than 3, were excluded. Patients who were smokers or taking angiotensin-converting enzyme inhibitors (ACEI) were identified. Patients with apparent purulent sputum or purulent nasal or postnasal discharge or bloody sputum were also excluded from this study. The remaining patients were divided into two groups for empirical trials of PNDS, asthma and/or GERD therapy, with two weeks for one trial of therapy: Group A, patients with typical symptoms or signs of PNDS, asthma syndrome (AS) and/or GERD; Group B, patients with no typical symptoms or signs. In Group A patients, the clinically probable diseases were first treated according to the typical symptoms or signs (if more than one probable diseases they were treated simultaneously). Relevant diseases were diagnosed when improvement was observed; otherwise, the remaining disease was treated in the sequence of PNDS, AS and GERD. PNDS was first treated in Group B patients. PNDS was diagnosed when improvement was observed; otherwise, the remaining disease was treated in the sequence of AS and GERD. Patients unresponsive to all trials in Groups A and B underwent available tests, such as parasinus or lung spiral CT (Toshiba Aquilion 64 CT, Japan), spirometry (PROFILER DX.830501-206, USA) with a bronchodilating test, bronchoscopy (Olympus BF 1 T180, Japan) and gastroscopy (Olympus, Japan). Patients continued to maintain daily cough diaries to evaluate daily cough scores [11]. An improvement was defined as increase in score (≥ 2) and improvement of cough by 50% or more. When these criteria are met, we diagnose the relevant disease.

### Typical symptoms, signs, and empirical trials of therapy

**PNDS:** Symptoms and signs: Nasal or postnasal discharge (by history or physical examination), repetitive throat clearing, a cobblestone appearance of the posterior pharyngeal mucosa, nasal obstruction or congestion, facial pain, frequent or repetitive sneezing, runny nose, itchy nose, significant allergens and irritants.

Treatment: Avoidance of allergens and irritants; first-generation antihistamines; decongestants [compound pseudoephedrine HCl sustained release capsules (SK&F, Tianjin, China), one tablet, once or twice a day or chlorphenamine maleate tablets, 4 mg, one to three times a day and pseudoephedrine hydrochloride tablets, 30 mg to 60 mg, three times a day]; and topical intranasal corticosteroids (budesonide nasalspray, 64 μg per nostril, twice a day) for two weeks [for allergic rhinitis, second-generation antihistamines preferable, such as loratadine (Schering-Plough, Shanghai, China), 10 mg, once daily].

**Asthma Syndromes (AS)** (including cough prominent asthma, CVA and EB). Symptoms and signs: Seasonal cough, history of asthma, significant atopy, wheezing, tightness in the chest, shortness of breath, cough at night, highly suspected allergens and irritants in the house, working place and so on.

Treatment: Avoidance of allergens and irritants; short acting β_2_ agonists, one puff to two puffs [terbutaline inhaler (AstraZeneca, England), 0.25 mg to 0.5 mg or albuterol inhaler (GlaxoSmithKline, England), 100 μg to 200 μg], three to four times a day; inhaled corticosteroid [budesonide (AstraZeneca, England), 400 μg, two times a day] or fluticasone/salmeterol 50/250 (GlaxoSmithKline, England), one puff, two times a day. After one week, if no improvement was noticed, 30 mg/d to 40 mg/d of prednisone was added for another week.

**GERD:** Symptoms and signs: Heartburn, regurgitation, epigastric distension, dyspepsia, chest pain (ruling out heart diseases), cough occurring after or during meal.

Treatment: Light meals three times a day; no food or beverage between meals except for water. Do not eat or drink 2 h to 3 h before lying down except for medications. Elevate head of bed. Omeprasole (AstraZeneca, England), 40 mg, twice a day (morning and evening before meal), and prokinetic agents three to four times a day, before meals and bedtime [domperidone (Xian-Janssen Pharmaceutical Ltd., China) 40 mg/d to 80 mg/d], two weeks for the treatment.

### Statistical analysis

Data were expressed as mean ± standard deviation. Student’s *t* test was used to compare quantitative data, and chi-square test was used to compare qualitative data. Statistical significance was set at p < 0.05. All reported probability values were two-sided.

## Results

A total of 700 qualified patients with chronic and subacute cough were recruited for study. Among the 700 patients, 42 (6.0%) had smoker’s cough and 30 (4.3%) were taking ACEIs. The remaining 628 patients were assigned to either Group A (324 patients) or Group B (304 patients) for empirical trials of therapy. In Groups A and B, 12.65% (41/324) and 20.72% (63/304) of the patients, respectively, were lost to follow up. Thus, 283 patients in Group A and 241 patients in Group B were available for further analysis. There was no difference between the cough scores in Group A (4.5 ± 1.4) and in Group B (3.9 ± 1.9) (p > 0.05). Among these 524 patients, 199 were males (38.0%) and 325 (62.0%) were females. The median of age was 39 years (between 18 and 72 years), and the median of cough duration was 14 months (3 weeks to 12 years).

In Group A, 153 patients improved at the first trial of therapy. Thus, the diagnosis rate of the first trial and the mean positive predictive value of the typical symptoms and signs were 54.1% (153/283). A total of 245 patients were diagnosed; thus, the diagnosis rate was 86.6% (245/283) in Group A. The mean time to diagnosis (MTD) was 13.1 ± 5.6 days. In Group B, 69 patients improved at the first trial of therapy. Thus, the diagnosis rate (28.6%, 69/241) in this group was significantly lower than in Group A (p < 0.01). At the second trial of therapy, 84 patients improved. At the third trial of therapy, 29 patients improved. The MTD was 23.4 ± 7.2 d, which was significantly longer than that of Group A patients (p < 0.01). A total of 182 patients showed improvement. Thus, the diagnosis rate was 75.5% (182/241), that is not statistically different from that of Group A (p > 0.05). 97 patients with no improvement accepted available laboratory investigations. A total of 44 patients had positive results: 9 patients had rhinosinusitis, 7 interstitial lung disease (2 pulmonary alveolar proteinosis, 1 sarcoidosis, 2 hypersensitivity pneumonitis and others). In addition, 7 had bronchial tuberculosis, 4 relapsing polychondritis, 3 bronchial amyloidosis, 3 bronchial tumour, 2 chronic obstructive pulmonary disease, 2 lung neoplasm, 2 mediastinal disease, 2 bronchial foreign body, 1 cardiac orifice polyp, 1 vocal cord polyp and 1 bronchiectasis. A total of 53 patients had negative results.

**Figure 1:**
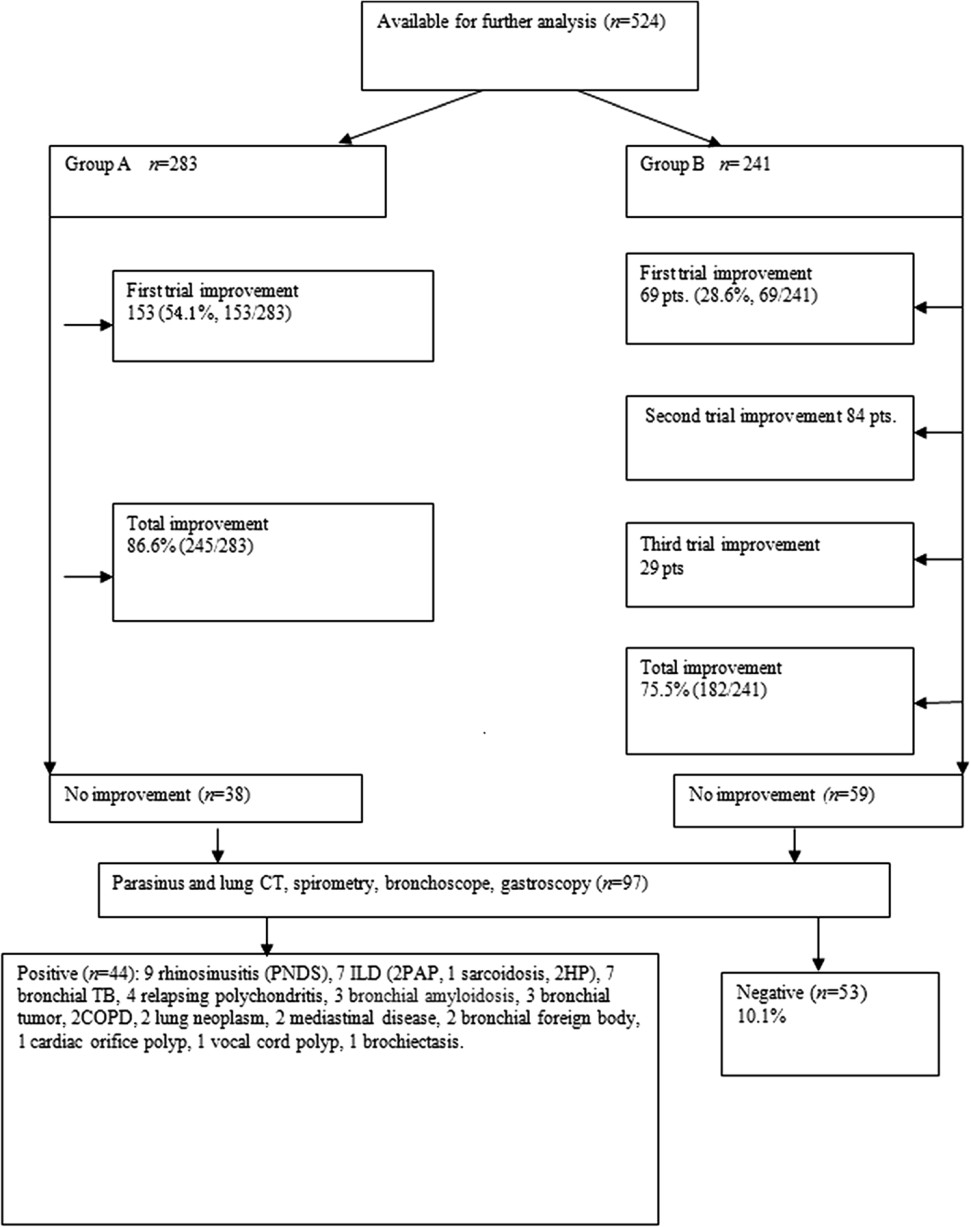
**Diagnostic algorithm.** COPD, chronic obstructive pulmonary disease; CT, computed tomography; HP, hypersensitivity pneumonitis, ILD, interstitial lung disease; PAP, pulmonary alveolar proteinosis.

Among 524 patients who accepted empirical trials of therapy and/or available laboratory investigations in Groups A and B, 436 (83.6%) were diagnosed with PNDS (179, including 9 patients diagnosed by CT (34.2%), AS (233, 44.5%) and/or GERD (53, 10.1%). Among these patients, 26 had two causes (26/436, 6.0%) and 6 patients had three causes (6/436, 1.4%). A total of 35 patients (35/524, 8.4%) were diagnosed as having other diseases, and 53 patients (10.1%) were left undiagnosed. After empirical trials of therapy, 81.5% of (427/524) patients were diagnosed (Table 1 and Figure 1).

**Table 1:** The causes of chronic and subacute cough

**PNDS**	**AS**	**GERD**	**Others**	**Undiagnosed**
34.2%	44.5%	10.1%	8.4%	10.1%

AS, asthma syndromes; GERD, gastroesophageal reflux disease; PNDS; postnasal drip syndrome. 26 patients with 2 causes, 6 patients with 3 causes.

## Discussion

PNDS, CVA, EB and GERD are the most common causes of chronic cough [17]. Patients with subacute cough that is not associated with an obvious respiratory infection were evaluated in the same way as patients with chronic cough [15]. Most cough patients with PNDS, CVA or EB are responsive to targeted therapy in one week to two weeks [17-20]. Cough patients with GERD improve in two weeks with high-dose omeprazole therapy [11]. Currently, many management strategies are available for cough; however, balancing the cost with time to treatment success is challenging [3]. Therefore, we designed a short-term diagnostic algorithm for patients with chronic and subacute cough; this algorithm focuses on empirical trials of PNDS, asthma and GERD therapy [3,19,21]. We also treated patients differentially. One group with suggestive symptoms and/or signs was treated consequently, whereas another group with no suggestive symptoms was treated sequentially.

Among the patients included in this study, 6.0% were smokers and 4.3% were taking ACEIs. Therefore, the smoking history and relevant medications of patients should be carefully reviewed. With this algorithm, we preliminarily diagnosed 84.3% patients with PNDS (34.2%), AS (51.1%) and/or GERD (10.1%) without employing 24 h oesophageal pH monitoring (24 h OpHM), bronchial provocation test (BPT) and induced sputum analysis (IS). Approximately 5.4% of the patients were diagnosed to have other diseases; only 10.1% patients were left undiagnosed (including 6 lost patients). Lai et al. [22] obtained similar results in a prospective, multi-centre survey in China, but we had more PNDS (34.1% vs 18.6%) and GERD (10.1% vs 4.6%) patients, due to the cold weather in Heilongjiang, the Northeastern province in China**,** comparing with the South warmer area in China.

Approximately, 81.5% of patients were diagnosed only with the empirical trials of therapy within six weeks. Even in Group B patients, who had no typical symptoms and signs, 75.5% were diagnosed. Only 10.1% of the patients needed further investigations through 24 h OpHM, BPT and IS. These results showed that the empirical trials of therapy were crucial in diagnosing the causes of chronic and subacute cough, whereas 24 h OpHM, BPT and IS had limited clinical usefulness [23,24]. PNDS, AS and GERD were the leading causes of chronic and subacute cough aside from post-infectious cough. Therefore, we should focus on these diseases when empirically treating patients. In addition, delaying laboratory tests is desirable for patients irresponsive to empirical trials of therapy [25]. The results of this study proved the proposed algorithm to be practical because it reduced the number of investigations performed and minimised unnecessary delays in treatment.

The mean positive predictive value of the typical symptoms and signs was 54.1%, that is comparable with the results of Ma [17] and Simpson [26] but considerably lower than that of Kastelik [27]. In Groups A and B, the diagnosis rates were 54.1% and 28.6% on the first trial, respectively (p < 0.01). The mean time for diagnosis in Group A (13.1 d) was significantly shorter than (23.4 d) in Group B (p < 0.01). The targeted therapy to the patients with suggestive symptoms and/or signs was better than the sequential one. Therefore, familiarisation with the typical symptoms and signs, carefully reviewing the history and performing physical examination are necessary for targeted treatments [3,6]. These procedures promote the improvement of many patients with empirical trials of therapy in a shorter time.

Further follow up was not performed in patients who showed improvement, which might underestimate multiple causes. The patients should be followed up to confirm the diagnosis and/or add a new diagnosis with additional trials and/or investigations when needed, which is beyond the purpose of this study. Many patients in Group A were treated for multiple causes simultaneously. Thus, we also might overestimate multiple causes in Group A. We did not differentiate between CVA and EB, which is needed for the purpose of research. Although CVA and EB have some differences, they have the same first line of therapy, i.e., corticosteroids. They also have a similar prognosis. However, whether EB represents a distinct clinical entity or shares a pathophysiological spectrum with CVA it remains to be elucidated [3,18]. Clinically, diagnosing them as one syndrome is feasible and practical; this strategy is comparable with the suggestion of He [28] that idiopathic interstitial pneumonia can be simply classified as steroid therapy-sensitive and steroid therapy-insensitive groups. Moreover, the primary objective for the patient is to eliminate the most disruptive symptom as quickly as possible [29].

Many patients lacked compliance. Longer and/or more expensive trials mean more patients lost to follow up. Therefore, shortening the trials and educating the patients are imperative [29]. This study suggests that treating AS first [3] or AS and PNDS [30,31] simultaneously might be an alternative approach to the diagnosis of patients without typical symptoms or signs. However, further studies must be conducted for verification.

## Conclusions

In conclusion, PNDS, AS and GERD are the major causes of chronic and subacute cough, except for post-infectious cough. This novel algorithm, which pays more attention to targeted or sequential empirical trials of therapy, resulted in a high diagnosis rate. Delaying laboratory tests is desirable for patients irresponsive to empirical trials of therapy. In addition, the targeted therapy is significantly better than the sequential therapy. Therefore, this algorithm would be a promising and practical approach to the diagnosis of chronic and subacute cough patients, especially in hospitals that lack some major diagnostic tests.

## Competing interests

The authors declare that they have no competing interests.

## Authors’ contributions

CJ: Design the study, apply for the registration number of the prospective clinical trial, treat patients and collect the data, write the paper. PL: Treat patients and collect the data. DZ: Treat patients and collect the data.
